# Multiple small tumor formation on both surfaces of the aortic valve cusps in Epstein–Barr virus-associated T/NK-cell lymphoproliferative disease: a case report

**DOI:** 10.1007/s11748-021-01613-5

**Published:** 2021-03-13

**Authors:** Ryuta Tai, Hiroyuki Irie, Yusuke Kinugasa, Hideki Teshima, Masahiko Ikebuchi, Keiko Kaneko, Nobuhiro Miyazaki, Hideaki Enzan, Tadashi Yoshino

**Affiliations:** 1 Cardiovascular Surgery, Chikamori Hospital, 1-1-16 Ohkawasuji, Kochi, 780-8522 Japan; 2 Neurology, Chikamori Hospital, 1-1-16 Ohkawasuji, Kochi, 780-8522 Japan; 3 Radiology, Chikamori Hospital, 1-1-16 Ohkawasuji, Kochi, 780-8522 Japan; 4 Diagnostic Pathology, Chikamori Hospital, 1-1-16 Ohkawasuji, Kochi, 780-8522 Japan; 5 Department of Pathology, Okayama University Graduate School of Medicine, Dentistry and Pharmaceutical Sciences, 2-5-1 Shikata-cho, Kita-ku, Okayama, 700-8558 Japan

**Keywords:** Aortic valve, Epstein–Barr virus, Cardiac tumor, Lymphoproliferative disease

## Abstract

A 41-year-old woman presented acute cerebral infarction. Transesophageal echocardiography revealed multiple masses only on both surfaces of the aortic valve cusps. There was no primary lesion outside the heart according to various examinations. After treatment for cerebral infarction, we replaced the aortic valve instead of preservation because the intraoperative histological examination reported that malignancy was highly suspected. Contrary to the rapid frozen section diagnosis, histological and immunohistochemical examinations failed to exhibit malignancy. The tumors were composed of atypical large lymphoid cells and they were assessed to be related to T-/natural killer-cells. Furthermore, Epstein–Barr virus related markers were also positive. Her three-year postoperative course was uneventful without chemotherapy. We report an extremely rare case of Epstein–Barr virus-associated T-/natural killer-cell lymphoproliferative disease which formed multiple small tumors on both surfaces of the aortic valve.

## Introduction

Epstein–Barr virus (EBV) is quite the common virus. EBV has been involved in the occurrence of a wide range of not only B-cell lymphoproliferative disorders (LPD) but also T/NK-cell lymphomas. These LPD entities often pose diagnostic challenges, both clinically and pathologically, because LPD widely contains clinically silent, benign lymphocytes and malignant lymphoma. We report an extremely rare case in which EBV-associated cytotoxic T/NK-cell LPD (EBV–T/NK LPD) formed aortic valve tumors.

## Case

A 41-year-old, otherwise healthy, woman suddenly experienced aphasia and was admitted in our hospital. Her body temperature was 38 °C. We could not find any abnormal manifestations except for sore throat. She had no previous diseases that were noteworthy. Magnetic resonance imaging showed a single lesion of large fresh cerebral infarction in the left temporo-occipital lobe (5 cm × 3 cm in size). MRA showed no proximal stenosis and less left M2 branches. It seemed to be a cerebral embolism. Transesophageal echocardiography revealed multiple small tumor masses on both surfaces of the aortic valve cusps. The tumors were loosely attached to both sides of the three aortic valve cusps and moved in association with her heartbeat, mimicking a pendular movement. Aortic valve regurgitation was trivial. Laboratory results showed normal WBC count (7100/µl) and low CRP levels (0.1 mg/dL). Blood culture for bacterial infection was negative. Blood tests for virus infection, including HIV, HTLV-1, HBV, and HCV, were all negative. Tumor markers such as AFP, CEA, CA19-9, NSE, SCC, CYFRA, CA15-3, and ProGRP were within normal limits. We preoperatively suspected that the tumor was a primary cardiac tumor such as papillary fibroelastoma. Although the tumor masses in the aortic valve cusps appeared to be fragile and had the possibility of leading to a recurrence of cerebral infarction, we waited for three weeks to reduce the risk of hemorrhagic infarction. Edaravone and argatroban hydrate were administered for the treatment of cerebral infarction. Anticoagulant therapy and antibiotic were not applied. Three weeks after the cerebral infarction, surgical operation was performed.

**Fig. 1:**
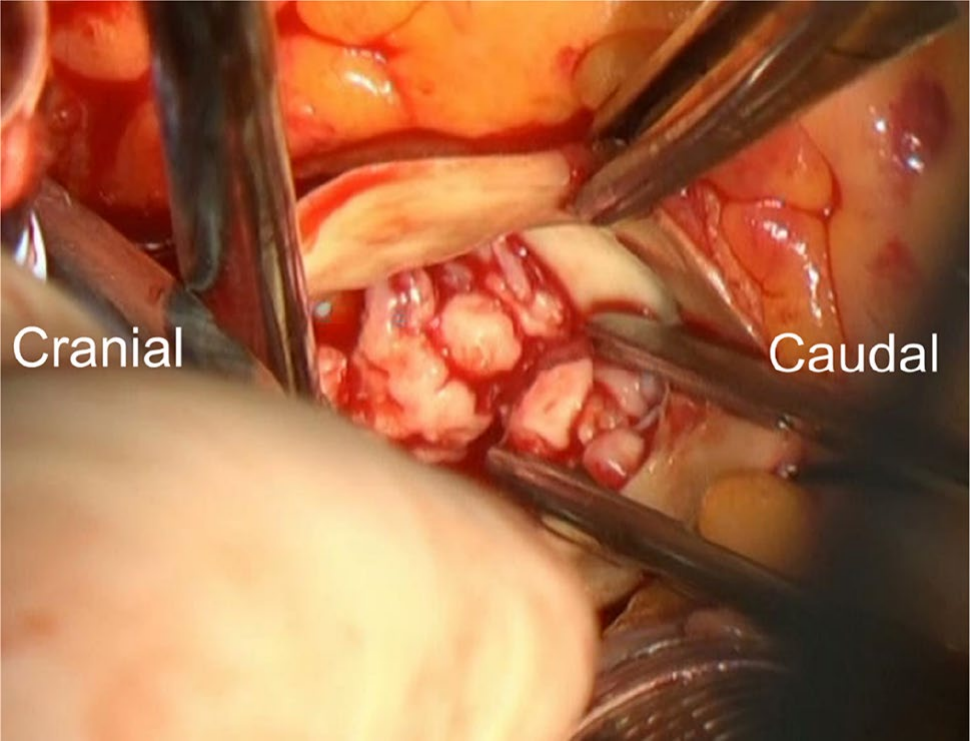
The valve and tumor appearance. They are whitish and fragile small tumors on the aortic valve cusps. The tumors are peeled off easily. Aortic cusps after removing tumors have no significant changes.

Macroscopically, small papillary tumors on the three aortic valve cusps were distributed irregularly in both left ventricular and aortic sides of the cusps. They were whitish and fragile (Fig. 1). All the tumors were peeled off easily from the aortic valve cusps. After tumor removal, all cusps had nearly normal appearance. The aortic wall, myocardium, and pericardial space looked intact. The tumors were then submitted for rapid frozen section diagnosis. The intraoperative histological examination revealed that the tumors were composed of atypical large lymphoid cells. They showed papillary growth. Since tumor malignancy was strongly suspected, we removed the cusps and replaced the valve with a Carpentier-Edwards PERIMOUNT Magna Ease pericardial aortic bioprosthesis with ThermaFix process, 21 mm (Edwards Lifesciences Corp, Irvine, California), instead of preservation. She chose bioprosthetic valve because she had desire to bear children. Postoperative course was uneventful, and she was discharged on the 9th postoperative day. Gallium scintigraphy, enhanced CT, PET-CT, upper gastrointestinal fiberscopy, colonoscopy, and bone marrow aspiration did not show any predominant tumors.

**Fig. 2:**
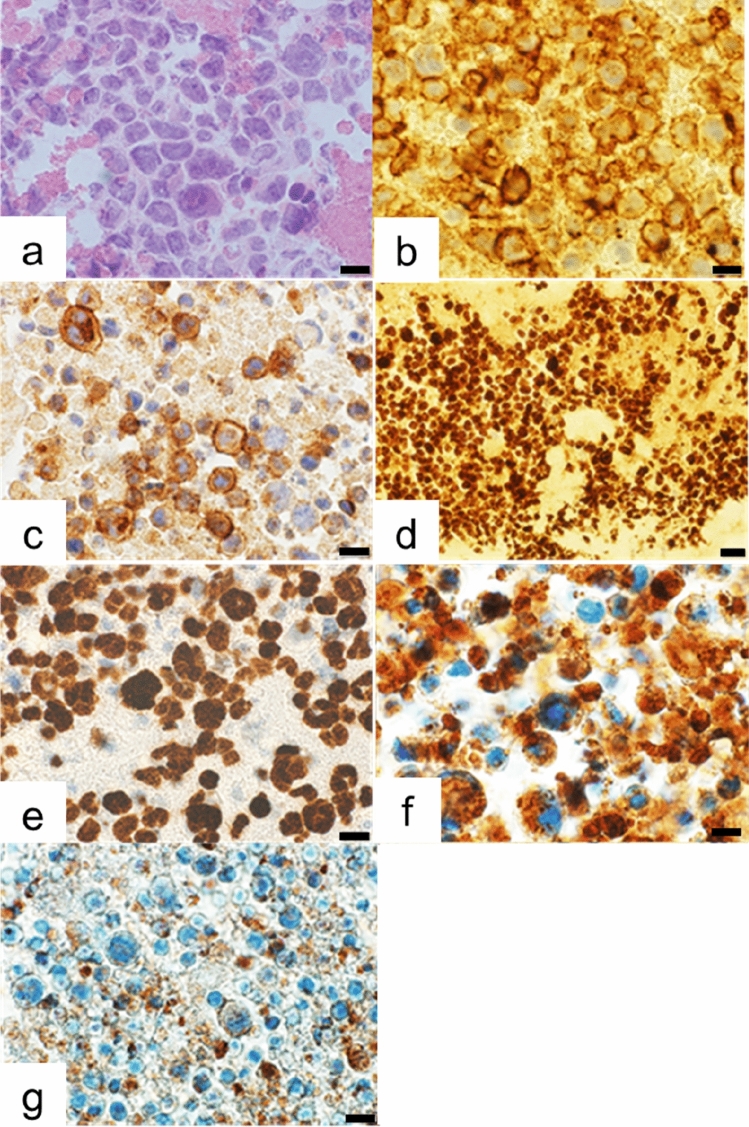
Histological and immunohistochemical examinations. Histological features of formalin-fixed and paraffin-embedded tumor tissues show dense infiltrations of atypical large lymphoid cells (**a**) (Hematoxylin–Eosin stain). They are positive for CD8 (**b**), CD30 (**c**), and the MIB-1 labeling index (**d**) is high. EBER in site hybridization highlights these atypical cells (**e**), while they are negative for CD56. Granzyme B (**f**) and TIA-1 (**g**) are positive. Barr: 10 µm (**a**, **b**, **c**, **e**, **f**, **g**), 20 µm (**d**)

After histological and immunohistochemical examinations of the tumor cells, it was found that the atypical large lymphoid cells (Fig. 2a) were positive for CD3, CD8 (Fig. 2b), and CD30 (Fig. 2c), and the MIB-1 labeling index was high (Fig. 2d). They were assessed to be related to T-/NK-cells. Furthermore, EBER-1 in situ hybridization (Fig. 2e) and LMP-1 were also positive, while CD20cy, CD4, CD5, ALK, and CD56 were negative. These results meant the tumor cells were infected by EBV. In general, CD8-positive cytotoxic T-cells and NK-cells contain cytotoxic granules in their cytoplasm, showing immunohistochemically positive reaction for granzyme B (Fig. 2f) and T-cell intracytoplasmic antigen (TIA-1) (Fig. 2g). We diagnosed this tumor to have originated due to EBV-T/NK LPD. She has had no signs of recurrence three years after the cardiac operation.

## Discussion

Primary cardiac tumors are relatively rare, being identified in less than 0.1% in pathologic autopsy series, and approximately 70% of them are reported to be benign [1, 2]. Malignant lymphoma (ML) is also rare [3]. In this case, intraoperative frozen section diagnosis of the tumors of the aortic valves suggested lymphoid malignancy. However, the immunohistochemical observation of the atypical large lymphoid cells suggested EBV-T/NK LPD. We could not find any clinical report that documented EBV-T/NK LPD forming in the aortic valve. Seventy-seven percent of cardiac papillary fibroelastoma arises from the valves [4]. Whereas, ML rarely arises from the valves. Motomatsu et al. reported a case of T-cell lymphoma on the mitral valve [5]. Each cardiac tumor has a preferred site on which to occur, but it is unclear why EBV-T/NK LPD formed on only the aortic cusps.

EBV is a ubiquitous herpes virus and exhibits tropism for B-cells, but in some cases, EBV infects T-cells, NK-cells, and even epithelial cells. Most EBV-infected patients are asymptomatic, but in some populations, EBV causes a variety of diseases such as infections, hematologic malignancies, and non-hematologic malignant diseases [6, 7]. It is known that LPDs occur in immunodeficiency patients or post-transplant patients. However, she was not immunocompromised. EBER-1 in situ hybridization and LMP-1 were positive in the cardiac tumor cells. It denoted EBV infection in the atypical large lymphocytes. Immunostaining showed that T/NK-cell markers were positive. EBV existed in the tumors, and infected T/NK-cells proliferated and formed the tumors on both surfaces of the aortic valve cusps.

EBV is associated with ML, and a lot of the cases involving ML undergo chemotherapy. There was a report for a case where only surgical resection for T-cell lymphoma was carried out [5]. However, ML has the possibility of recurrence without chemotherapy. In this case, she was diagnosed with EBV-T/NK LPD, which was different from ML, even though the MIB-1 labeling index was high. Although it was high, we considered that the tumors might not be malignant and the tumors were grossly and histologically resected to the extent possible. Therefore, chemotherapy was not performed. At present, there have been no signs of recurrence for over three years. The reason presumed was that EBV-T/NK LPD maintained normal immunocompetence resulting in a self-limiting course, which differed from ML.

This is an extremely rare case in which EBV-T/NK LPD formed tumors in the aortic valve.

## Conclusion

We experienced an extremely rare case; EBV-T/NK LPD formed tumors on only the aortic valve cusps. Intraoperatively, the tumor was highly suspected malignant, therefore, she underwent aortic valve replacement. At present, she has had no signs of recurrence for three years.
